# Graphene-nanodiamond membranes for atomic-impact sensing: design and fluorescence readout limits

**DOI:** 10.1039/d6na00363j

**Published:** 2026-09-17

**Authors:** Johannes Fiedler, Sabrina D. Eder, Justas Zalieckas

**Affiliations:** a Department of Physics and Technology, University of Bergen Allegaten 55 Bergen 5007 Norway johannes.fiedler@uib.no

## Abstract

Hybrid nanomaterial platforms that combine mechanically compliant two-dimensional membranes with optically active quantum emitters offer an attractive route towards ultrasensitive particle detection. Here, we investigate a sensor concept based on a graphene membrane suspended over a nanoscale aperture and functionalised with fluorescent nanodiamonds. The impact of an incoming atom transfers momentum to the membrane and excites an out-of-plane mechanical motion, which is transduced into an optical signal through the nanodiamond fluorescence. We develop a quantitative upper-bound model linking atomic momentum transfer, the mechanical response of the nanodiamond-loaded membrane, and the resulting optical transduction. Design maps for the mechanical resonance frequency and collision-induced oscillation amplitude reveal single-impact displacements in the femtometre-to-sub-picometre range for helium atoms. Even under favourable mechanical, collision, and optical conditions, the corresponding relative fluorescence modulation remains below 10^−6^ throughout the investigated parameter space and reaches only a few 10^−7^ under the most favourable conditions. Combined with photon-counting statistics, these results show that direct fluorescence-intensity detection of individual atomic impacts is impractical in the considered configuration. Importantly, the analysis identifies optical displacement transduction, rather than the absence of a mechanical response, as the principal limitation. The predicted absolute displacements approach the scale accessible to state-of-the-art graphene optomechanical measurements, making interferometric or cavity-based displacement readout a more promising direction, although single-impact detectability remains dependent on bandwidth and noise. Our results establish quantitative feasibility bounds for graphene-nanodiamond atomic-impact sensors and identify the readout mechanism as the central design parameter for future hybrid nano-mechanical sensing architectures.

## Abstract 2

Hybrid nanomaterial platforms that combine mechanically compliant two-dimensional membranes with optically active quantum emitters offer an attractive route towards ultrasensitive particle detection.

## Graphical abstract

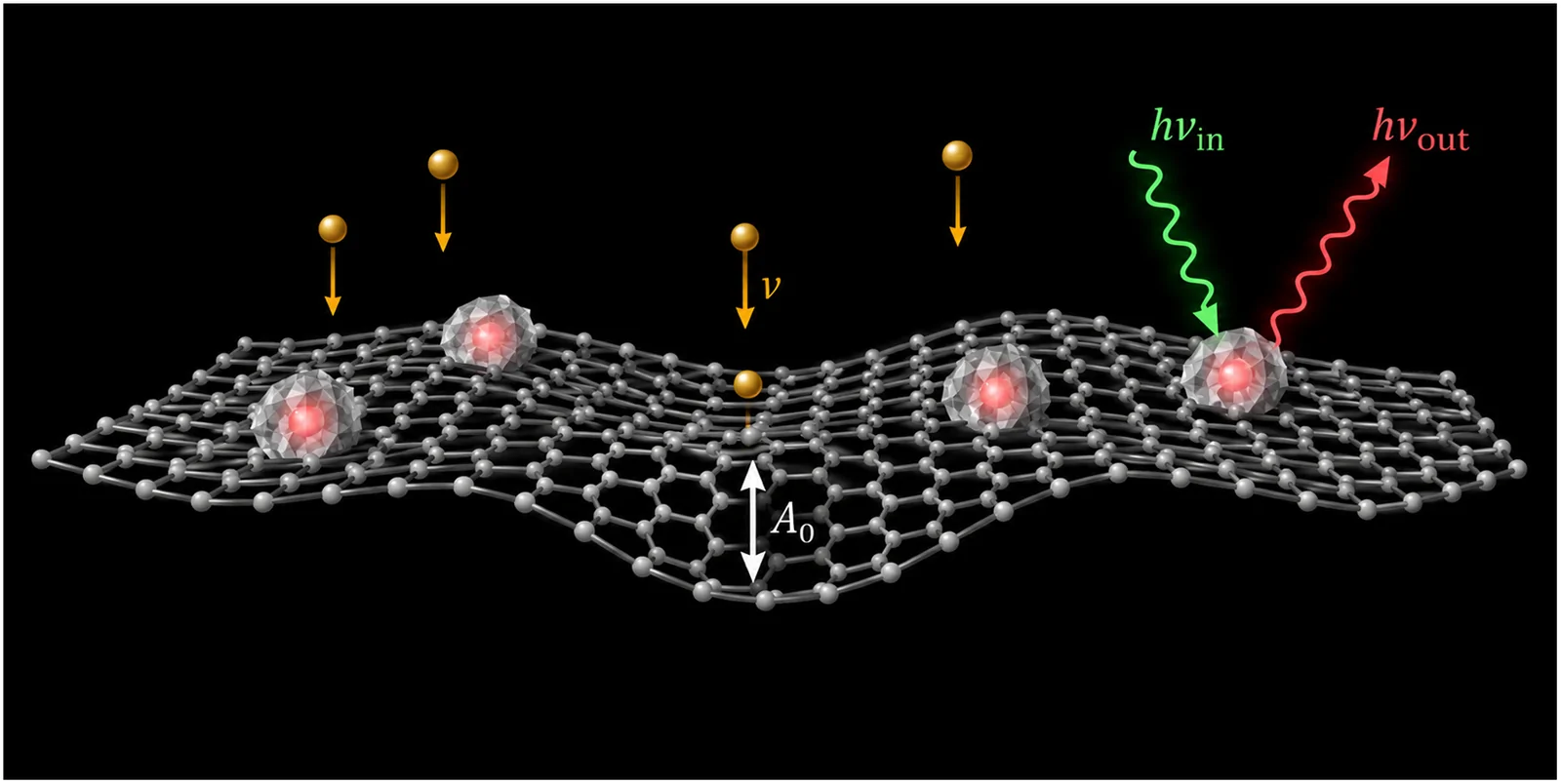

## 1 Introduction

The detection of individual particles interacting with nanostructures plays an important role in a wide range of fields, including atomic physics,^1–3^ nanoscale sensing,^4–6^ and quantum technologies.^7–11^ In particular, the ability to detect atomic impacts with high spatial and temporal resolution is relevant for applications such as atomic beam diagnostics,^12^ matter-wave interferometry,^13^ and studies of atom-surface interactions.^14,15^ Over the past decade, nanoscale mechanical resonators have emerged as promising platforms for detecting extremely small forces and masses due to their low effective mass and high mechanical quality factors.^16,17^

Two-dimensional materials provide a particularly attractive platform for such nano-mechanical sensors. Graphene membranes combine extremely low areal mass density with high mechanical strength and can sustain large elastic deformations while maintaining high resonance frequencies.^18,19^ Suspended graphene resonators have therefore been widely investigated as ultrasensitive mechanical elements for mass sensing, force detection, and pressure measurements.^20,21^ The combination of low mass density and high mechanical stiffness makes graphene membranes particularly responsive to weak external mechanical perturbations.

In parallel, optically active defects in diamond have emerged as versatile nanoscale probes for sensing and quantum technologies. In particular, nitrogen-vacancy (NV) centres in diamond exhibit stable fluorescence and long spin coherence times even under ambient conditions, enabling applications ranging from nanoscale magnetometry to bioimaging.^22^ Nanodiamonds containing NV centres can be integrated into a wide variety of hybrid nanostructures, providing an optical interface for mechanical and environmental sensing. Hybrid graphene-nanodiamond NEMS have already demonstrated electromechanical coupling between graphene motion and NV-centre emission,^23^ providing experimental motivation for combining these material platforms.

Combining these two material platforms suggests a hybrid sensor architecture in which a suspended graphene membrane serves as the mechanical sensing element, while nanodiamonds act as optical reporters of membrane motion. In such a configuration, the impact of an incoming atom transfers momentum to the membrane and excites a transient mechanical vibration that can, in principle, modulate the fluorescence signal of the nanodiamond.

Despite the conceptual simplicity of this approach, it remains unclear whether the momentum transferred by a single atomic collision is sufficient to generate an experimentally resolvable signal. This requires connecting three distinct stages of the transduction process: the atomic momentum transfer, the resulting mechanical displacement of the nanodiamond-loaded graphene membrane, and the optical transduction of this displacement. A quantitative feasibility bound linking these stages has, to our knowledge, not been established for graphene-nanodiamond membranes.

In this work, we develop a quantitative upper-bound model of atomic-impact sensing using nanodiamond-functionalised graphene membranes. We first determine the resonance properties of the loaded membrane and the displacement generated by an individual atomic collision as functions of membrane geometry, nanodiamond loading, and collision energy. We then propagate this mechanical response through a fluorescence-based optical readout model and assess the resulting signal against fundamental photon-counting constraints. Finally, we compare the predicted absolute displacements with reported sensitivities of alternative graphene optomechanical readout schemes. The resulting design maps identify which stage of the transduction chain limits single-impact detection and how this limitation scales with the relevant device parameters.

In particular, we focus on helium atoms as a representative and stringent test case. Their low atomic mass limits the available collision momentum at a given kinetic energy, making helium particularly challenging for mechanically mediated impact detection. While even lighter species such as hydrogen could in principle be considered, their increased chemical reactivity opens additional detection pathways that fall outside the purely mechanical sensing mechanism investigated here.

Our results show that, even under favourable mechanical, collision, and optical conditions, the relative fluorescence modulation generated by a single helium impact remains below 10^−6^ throughout the investigated parameter space. Photon-counting statistics therefore make direct single-impact detection through fluorescence modulation impractical for the considered configuration. The analysis identifies the optical displacement transduction, rather than the absence of a mechanical response, as the principal bottleneck. The predicted femtometre-to-sub-picometre mechanical displacements motivate comparison with interferometric and cavity-based readout schemes, which have demonstrated substantially higher displacement sensitivities in graphene NEMS.^24–26^ These results establish quantitative feasibility bounds and provide design criteria for future hybrid nano-mechanical atom sensors.

## 2 Modelling

We consider a hybrid sensor consisting of a suspended graphene membrane spanning a single square aperture in a supporting substrate and decorated with fluorescent nanodiamonds, as illustrated in Fig. 1. A single incoming atom transfers momentum to the membrane, thereby exciting an out-of-plane mechanical motion. The resulting transient displacement is converted into an optical signal *via* the nanodiamonds' fluorescence response.

**Fig. 1:**
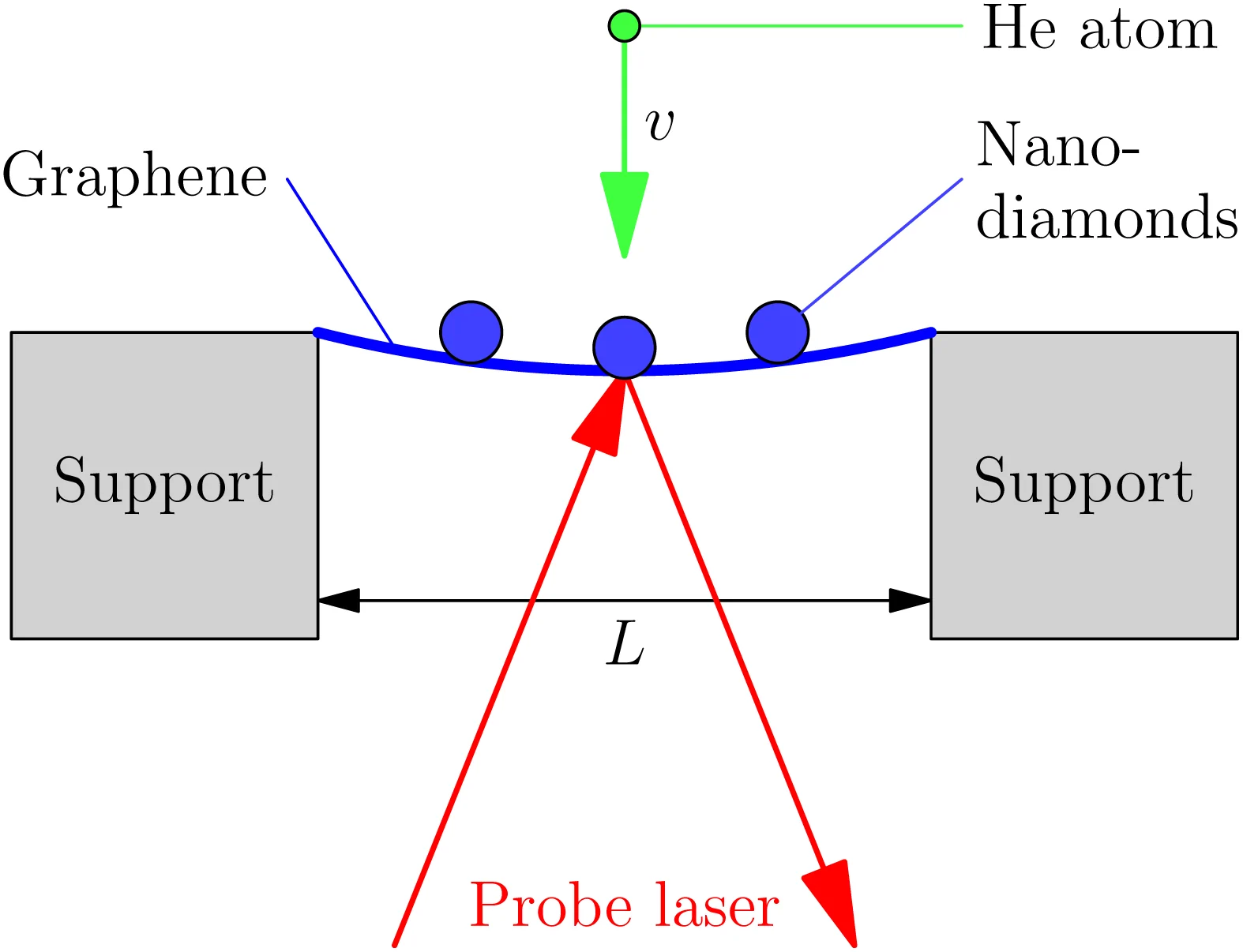
Schematic representation of the proposed hybrid sensor. A graphene membrane spans a square aperture in a supporting substrate and is functionalised with fluorescent nanodiamonds. An incoming atom transfers momentum to the membrane and excites an out-of-plane vibration, which can be probed optically through the fluorescence response of the nanodiamond layer.

In the following, we develop a minimal model for this process. We first determine the mechanical resonance of the graphene membrane and its dependence on the aperture size and nanodiamond loading. We then relate the momentum transfer of a single atomic collision to the amplitude of the excited membrane motion. Finally, we describe how this motion is converted into a fluorescence signal and evaluate its detectability. Throughout the analysis, we deliberately adopt favourable mechanical and optical assumptions in order to establish an upper bound on the achievable sensor response. Additional dissipation, structural disorder, non-ideal optical collection, and detector noise would therefore reduce the experimentally accessible signal relative to the values obtained below.

### 2.1 Oscillation of a graphene sheet

We model the suspended graphene sheet as a square membrane of side length *L* under an effective in-plane tension *T*. Here, *T* denotes the effective residual tension of the suspended membrane and therefore incorporates fabrication-dependent pre-strain rather than representing a universal graphene material parameter. In the tension-dominated regime, the flexural eigenfrequencies are approximated by^18,20^1
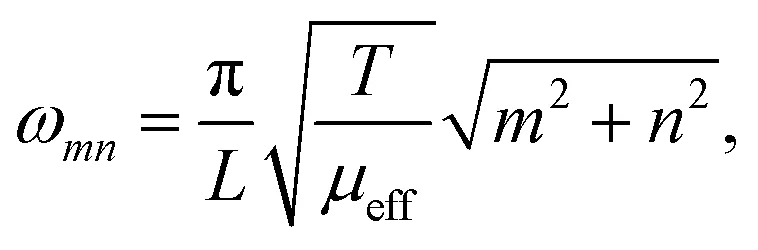
where (*m*, *n*) denote the membrane mode indices and *µ*_eff_ is the effective areal mass density. Restricting ourselves to the fundamental mode (*m*, *n*) = (1, 1) yields2
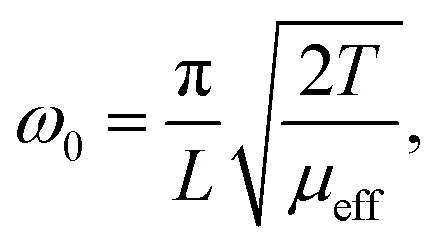
and3
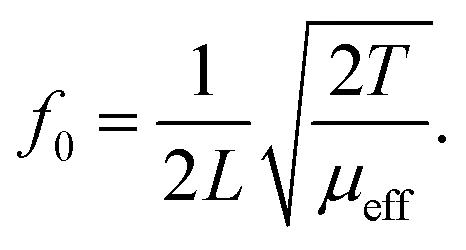


Eqn (3) assumes that the restoring force is dominated by membrane tension rather than bending rigidity. The relative importance of both contributions is characterised by *TL*^2^/*D*, where *D* denotes the bending rigidity. For monolayer graphene, Wei *et al.* reported *D* = 1.44 eV = 2.31 × 10^−19^ N per m.^27^ With the effective tension *T* = 0.10 N per m used here, even the smallest aperture considered, *L* = 200 nm, gives *TL*^2^/*D* ≃ 1.7 × 10^4^ ≫ 1. The complete parameter range of Fig. 2 therefore lies well within the tension-dominated regime.

**Fig. 2:**
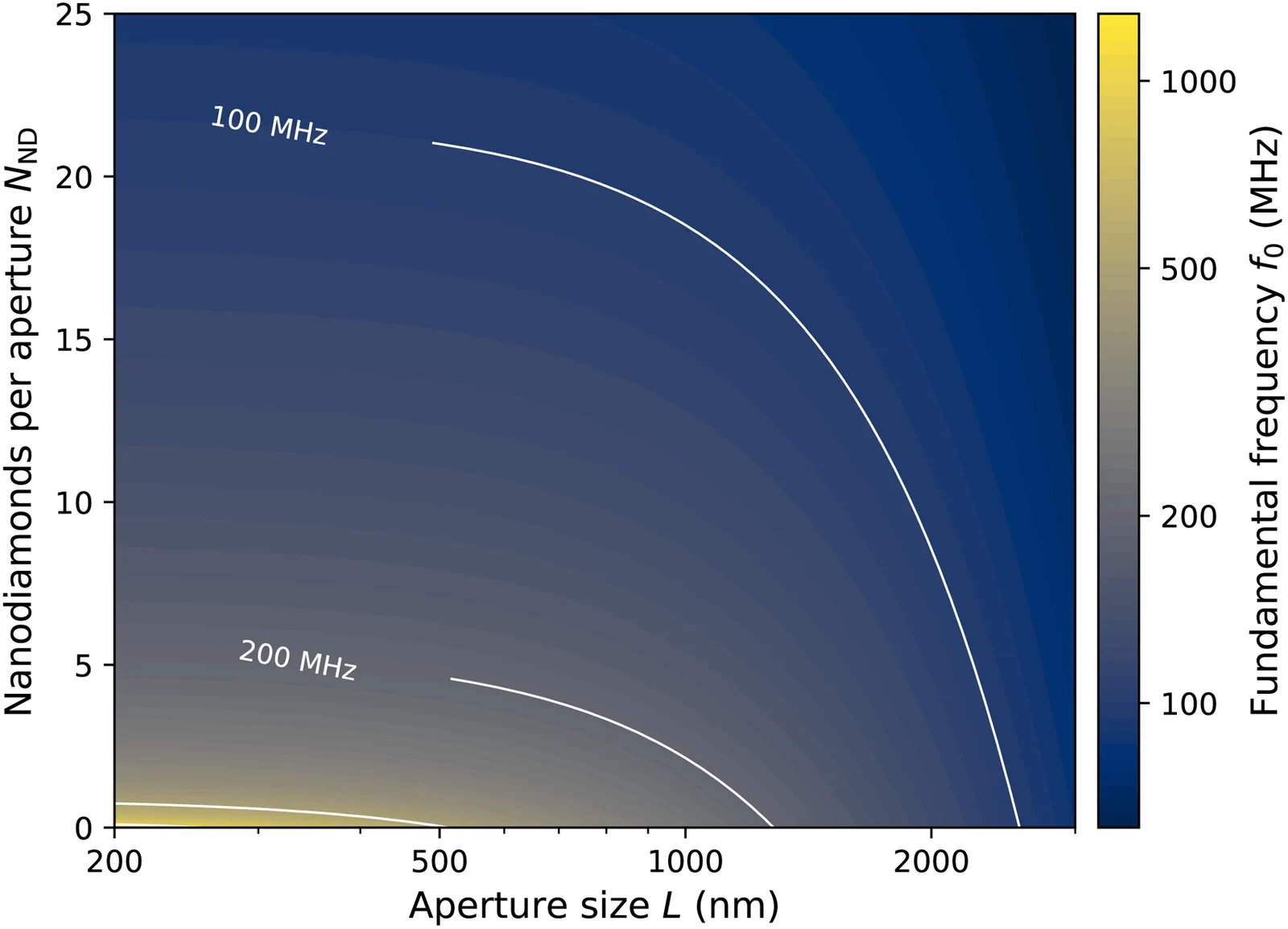
Fundamental resonance frequency of the graphene membrane as a function of aperture size *L* and nanodiamond loading *N*_ND_. The calculations assume an effective membrane tension *T* = 0.10 N per m, a graphene areal mass density *ρ*_g_ = 7.6 × 10^−7^ kg m^−2^, and spherical nanodiamonds with radius *R*_ND_ = 25 nm and density *ρ*_ND_ = 3.5 × 10^3^ kg m^−3^. Increasing the aperture size reduces the resonance frequency, whereas additional nanodiamond loading lowers it due to the increased effective mass density.

The effective areal mass density contains contributions from both the graphene membrane and the nanodiamond coating,4
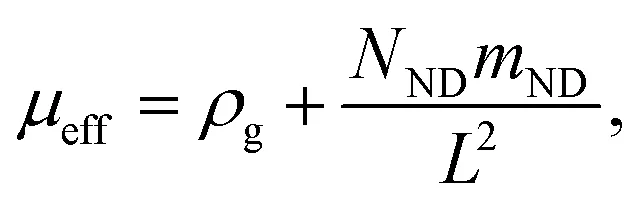
where *ρ*_g_ is the areal mass density of graphene, *N*_ND_ the number of nanodiamonds within one aperture and *m*_ND_ the mass of a single nanodiamond. The graphene areal density is approximately *ρ*_g_ ≈ 7.6 × 10^−7^ kg m^−2^.^19^ Approximating the nanodiamonds as spheres of radius *R*_ND_ and density *ρ*_ND_ gives5
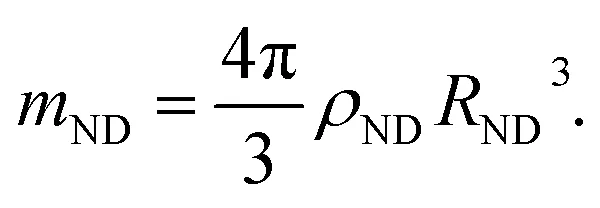


The effective modal mass of the fundamental mode is written as

6 *M*_eff_ = *ηµ*_eff_*L*^2^,

where *η* is a geometric mode factor of order unity describing the spatial mode profile.

In the present model, the nanodiamond layer is treated as an effective homogeneous mass loading. This approximation is justified as long as the nanodiamonds remain sparsely distributed compared to the membrane dimensions, such that the fundamental mode shape is only weakly perturbed. In particular, for discrete nanodiamond clusters that are small compared to the aperture size and do not form a continuous film, the dominant effect is an increase in the effective modal mass rather than a significant modification of the mode structure or boundary conditions. A real nanodiamond-functionalised membrane may additionally exhibit local strain, changes in residual tension, mode-shape perturbations, and additional mechanical damping. These device-specific effects are not included in the present homogenised description. Since the purpose of the model is to determine an optimistic upper bound on the collision-induced response, the homogeneous-loading approximation avoids introducing additional damping or structural disorder that would generally make detection less favourable.

Fig. 2 maps the resulting resonance frequency as a function of aperture size and nanodiamond loading, considering nanodiamonds with a radius of *R*_ND_ = 25 nm. For fixed effective areal mass density, eqn (3) gives the familiar membrane scaling *f*_0_ ∝ *L*^−1^. In the present map, however, *µ*_eff_ itself also depends on *L* for fixed *N*_ND_ through eqn (4). The variation with aperture size therefore reflects both the geometric *L*^−1^ dependence and the changing relative contribution of the nanodiamond mass loading. Increasing *N*_ND_ increases the effective mass density and consequently lowers the resonance frequency.

### 2.2 Single-atom collision

We consider the excitation of the membrane by the collision of a single atom of mass *m*_a_ and velocity *v* incident at an angle *θ* relative to the membrane normal. The transferred out-of-plane momentum is

7 *J* = *γm*_a_*v* cos *θ*,

where *γ* describes the effective momentum transfer depending on the scattering process. For specular reflection from a stationary surface, complete reversal of the normal momentum corresponds to *γ* = 2, whereas partial momentum transfer gives smaller values. Together with normal incidence, *θ* = 0, the choice *γ* = 2 therefore provides the largest impulse within the present collision model. We adopt this deliberately favourable limit in the calculations below; results for other collision scenarios follow directly from the linear scaling *J* ∝ *γ*  cos *θ*.

In terms of the atomic kinetic energy8
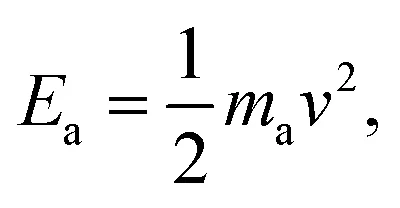
this can be written as9
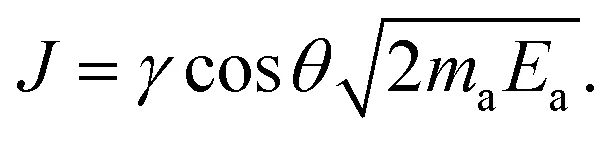


We further assume that the collision occurs at the centre of the membrane, where the displacement of the fundamental (1, 1) mode is maximal. For an impact at position (*x*, *y*), the excitation of this mode would acquire the corresponding local mode-shape factor and would therefore be smaller away from the membrane centre. The central-impact assumption therefore provides another upper bound for the single-event response.

Treating the membrane as a damped harmonic oscillator, the displacement following an impulsive collision is

10 *z*(*t*) =*A*_0_*e*^−*Γt*^sin(*ω*_d_*t*),

with a damping rate11
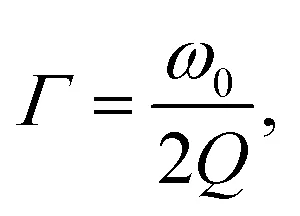
quality factor *Q*, and12
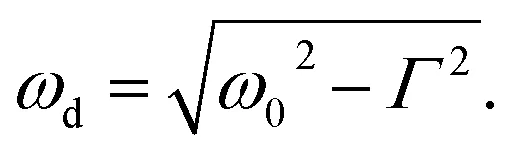


The initial oscillation amplitude is obtained from momentum conservation,13
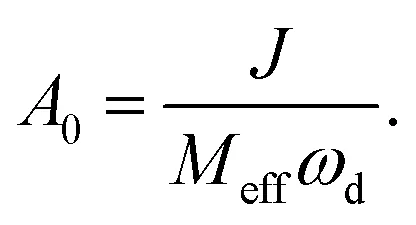


The quality factor controls primarily the duration of the subsequent ring-down rather than the initial displacement amplitude. In the underdamped regime *Q* ≫ 1,14
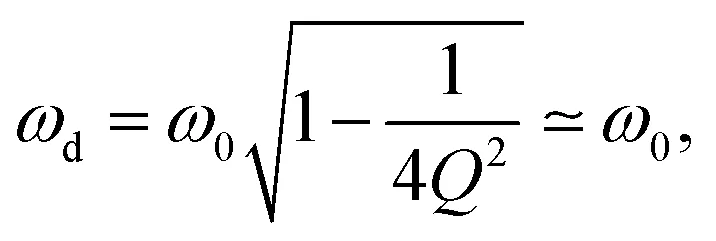
and hence15
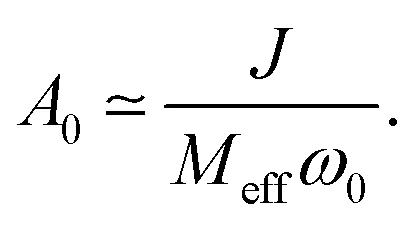


Thus, varying *Q* over the experimentally relevant underdamped regime has a negligible effect on the initial single-impact amplitude. Instead, *Q* determines the amplitude ring-down time16
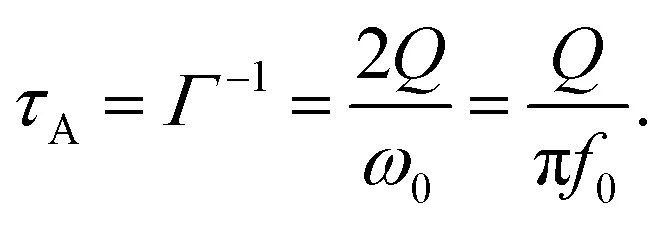


Combining eqn (3), (6), and (13) allows us to map the mechanical response to a single atomic impact. Fig. 3 shows the resulting oscillation amplitude for helium as a function of aperture size and atomic kinetic energy. The interval *E*_a_ = 0.1–200 meV encompasses the energy scale relevant to thermal and supersonic helium beams (typically a few to several tens of meV^28^) and extends beyond this regime to illustrate the energy dependence of the mechanical response. The interaction and transmission of helium atoms with atomically thin membranes in this energy range have also been investigated experimentally and theoretically.^29–31^ In the present model, we do not attempt to resolve the microscopic scattering dynamics. Instead, we assume the favourable limit of complete normal-momentum reversal, *γ* = 2, which provides an upper bound on the mechanically transferred impulse.

**Fig. 3:**
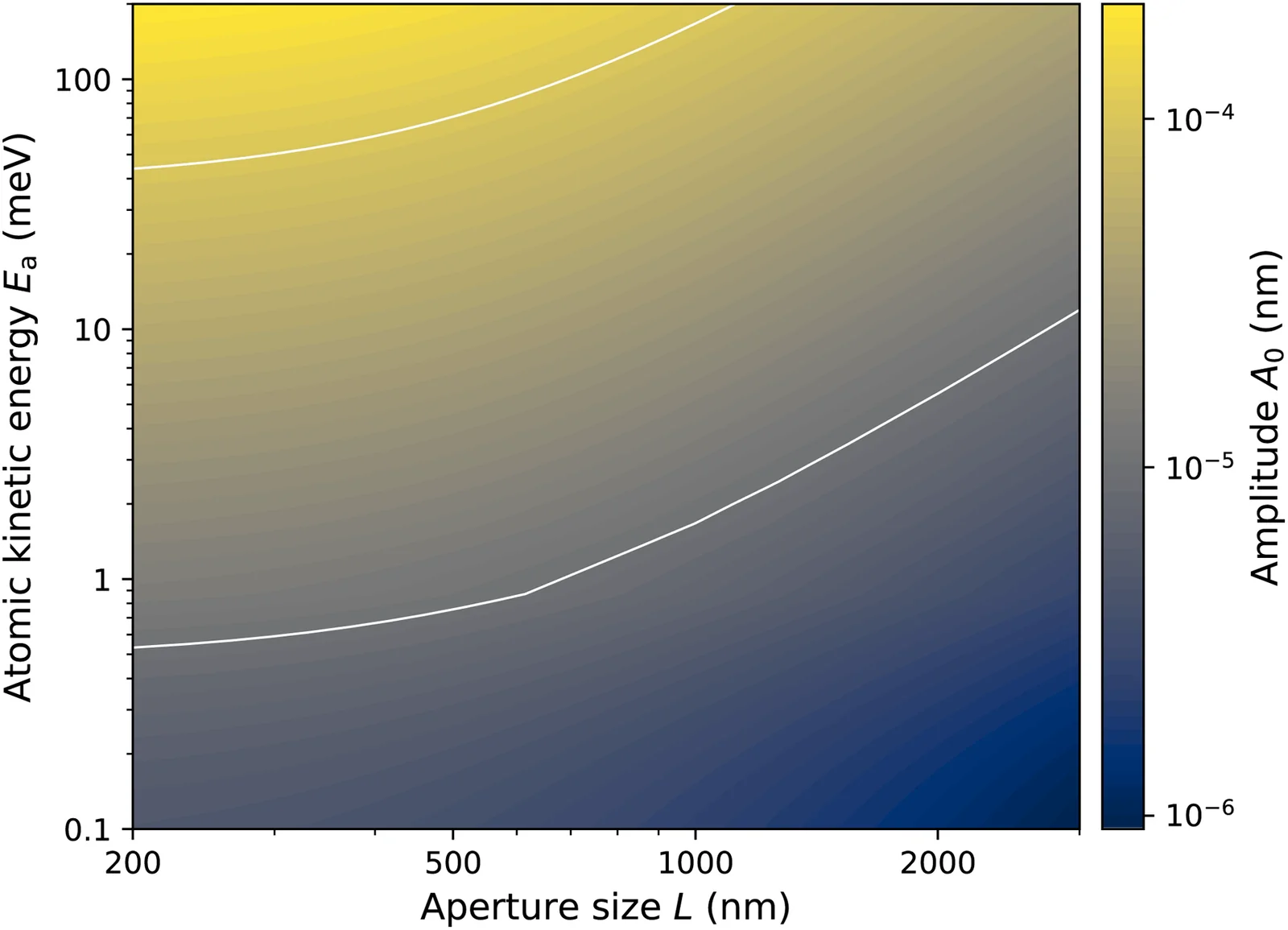
Peak oscillation amplitude *A*_0_ of the graphene membrane following a single helium impact as a function of aperture size *L* and atomic kinetic energy *E*_a_. The calculation assumes one nanodiamond per aperture with *R*_ND_ = 25 nm, an effective membrane tension *T* = 0.10 N per m, a modal mass factor *η* = 0.27, *Q* = 500, normal incidence (*θ* = 0), and complete momentum reversal (*γ* = 2). Even under these favourable conditions, the displacement remains well below the picometre scale throughout the considered parameter range.

Since 
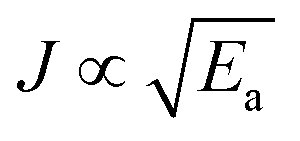
, increasing the kinetic energy produces only a square-root enhancement of the mechanical response. Even under the deliberately favourable conditions of normal incidence, complete momentum reversal, and impact at the maximum of the fundamental mode, the resulting displacement remains in the sub-picometre regime over the considered parameter space. Less efficient momentum transfer, oblique incidence, or off-centre impact can only reduce this response.

### 2.3 Fluorescence read-out

The membrane displacement is converted into an optical signal through the fluorescence of nanodiamonds attached to the graphene sheet. In a minimal model, the detected fluorescence count rate follows the axial point-spread function of the optical detection system,17
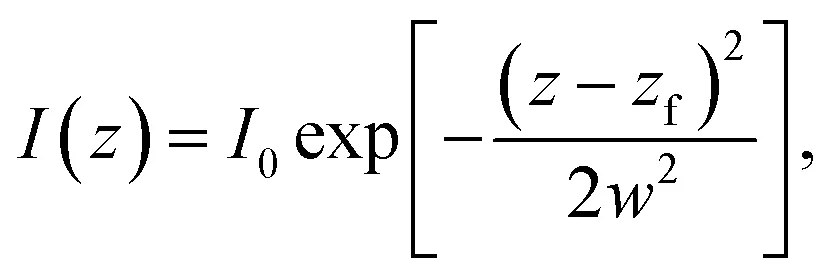
where *I*_0_ is the peak fluorescence rate, *z*_f_ denotes the centre of the detection profile, and *w* is the effective axial detection length.

For small membrane displacements *δz*, the fluorescence response can be linearised around an equilibrium position *z*_eq_, yielding18
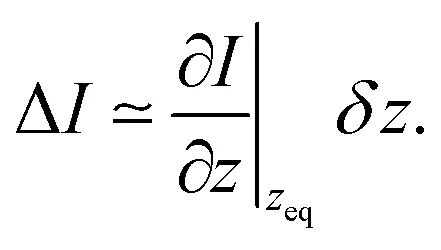


The magnitude of the slope of the Gaussian detection profile is maximal for |*z*_eq_ − *z*_f_| = *w*. Operating at this point therefore provides the largest possible linear fluorescence response, for which19
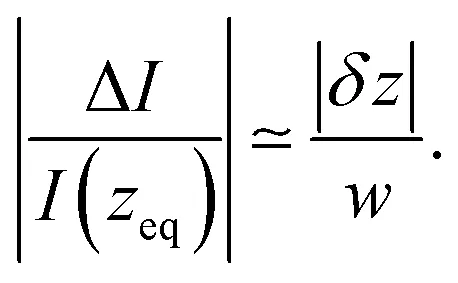


Accordingly, the dimensionless ratio *A*_0_/*w* provides an upper-bound measure of the fluorescence modulation generated by a single atomic impact.

Using the amplitudes obtained in Fig. 3, we obtain the map shown in Fig. 4. For an effective axial detection length of *w* = 300 nm, the predicted relative single-event modulation remains below 10^−6^ throughout the entire parameter range and reaches only a few 10^−7^ under the most favourable conditions.

**Fig. 4:**
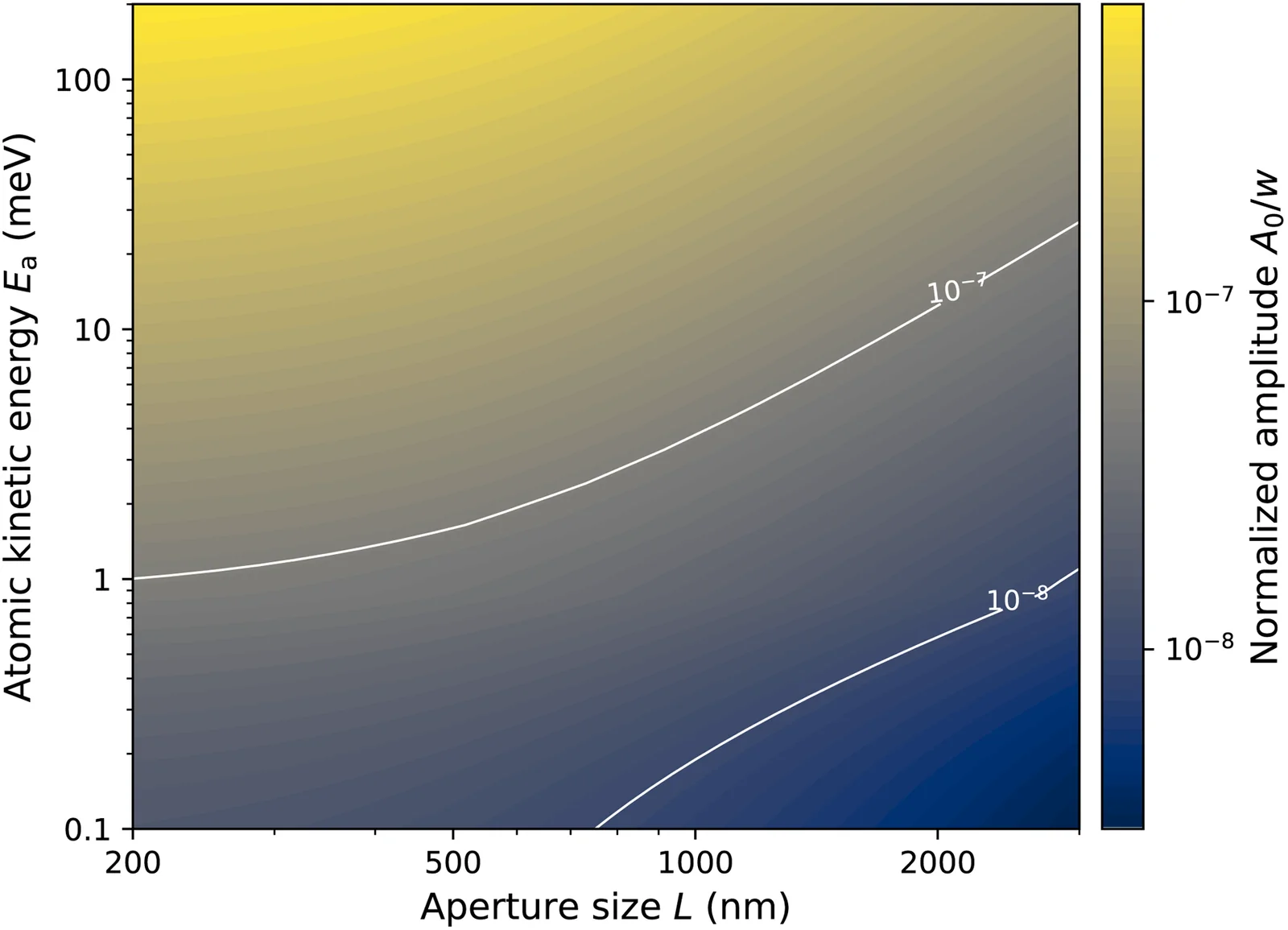
Normalised oscillation amplitude *A*_0_/*w* following a single helium impact, calculated from the mechanical response in Fig. 3 using an effective axial detection length *w* = 300 nm. The quantity *A*_0_/*w* represents the maximum linear relative fluorescence modulation within the Gaussian read-out model. Even under favourable collision and mechanical conditions, the single-event modulation remains below 10^−6^ throughout the considered parameter range.

Importantly, *A*_0_/*w* characterises the transduction amplitude rather than the signal-to-noise ratio of a particular experimental detector. We deliberately consider an idealised optical read-out in order to establish the most favourable limit of the proposed detection principle. Finite collection and detection efficiencies, background counts, detector noise, non-optimal emitter orientation, fluorescence quenching, and photobleaching would further reduce the experimentally accessible signal. In addition, even an otherwise ideal photon-counting measurement remains subject to photon shot noise. A quantitative signal-to-noise ratio would therefore depend on the detected photon number, integration time, and specific detection architecture and cannot be inferred from *A*_0_/*w* alone.

The present result consequently does not assign a universal minimum number of atoms required for detection. Instead, it establishes a detector-independent transduction limit: even under ideal mechanical, collision, and optical operating conditions, a single helium impact produces only a relative fluorescence modulation of order 10^−7^. Direct single-atom detection based solely on fluorescence-intensity modulation is therefore not feasible within the present architecture. The consequences for ensemble detection and alternative read-out strategies are considered below.

## 3 Discussion

The modelling results presented in the previous section provide a quantitative upper-bound assessment of atomic-impact detection using fluorescence read-out from nanodiamond-functionalised graphene membranes. The central result is summarised by Fig. 4. Even under favourable mechanical, collision, and optical conditions, the predicted relative single-event modulation remains in the range *A*_0_/*w* ∼ 10^−9^–10^−7^ over most of the considered parameter space and reaches only a few 10^−7^ under the most favourable conditions.

These values describe the transduction amplitude rather than the signal-to-noise ratio of a specific detector. Nevertheless, they already establish a stringent bound on direct fluorescence detection. For a photon-counting measurement containing *N*_*γ*_ detected photons, the relative shot-noise level scales as *N*_*γ*_^−1/2^. Resolving a relative modulation *ε* = *A*_0_/*w* with unit signal-to-noise ratio would therefore require, in the ideal shot-noise-limited case,

20 *N*_*γ*_ ≳ *ε*^−2^.

For *ε* ∼ 10^−7^, this corresponds to approximately 10^14^ detected photons. At the assumed peak fluorescence rate *I*_0_ = 10^6^ s^−1^, this photon number cannot be accumulated on the sub-microsecond to microsecond mechanical timescale of the single-impact response. Background counts, finite collection efficiency, detector noise, non-optimal emitter orientation, and fluorescence quenching would further reduce the experimentally attainable signal-to-noise ratio.

The limitation is therefore not associated with a particular choice of detection threshold but follows directly from the extremely small mechanical-to-optical transduction amplitude. In the following, we examine whether variations of the mechanical, collision, or optical parameters can substantially alter this conclusion.

### 3.1 Increasing the atomic momentum transfer

The single-impact response scales linearly with the transferred momentum,21
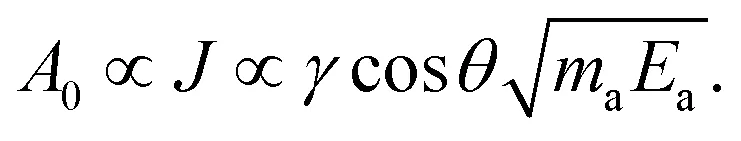


The calculations already assume the favourable limit *γ* = 2 and *θ* = 0, corresponding to complete reversal of the normal atomic momentum. Consequently, no enhancement is available from the collision geometry within the present model. Moreover, because 
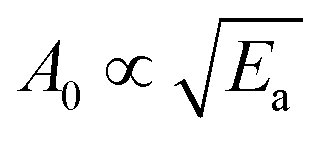
, increasing the response by a factor *g* through the kinetic energy alone requires an energy increase by *g*^2^. Even a two-order-of-magnitude enhancement of the mechanical amplitude would therefore require a four-order-of-magnitude increase in kinetic energy.

Heavier atoms could, in principle, increase the transferred momentum through the 
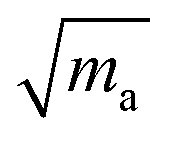
 scaling. However, this represents only a weak enhancement and would change the atomic species and hence the intended operating regime of the sensor.

### 3.2 Modifying the membrane geometry

For weak mass loading, the membrane model gives *M*_eff_ ∝ *L*^2^ and *ω*_0_ ∝ *L*^−1^. Consequently,22
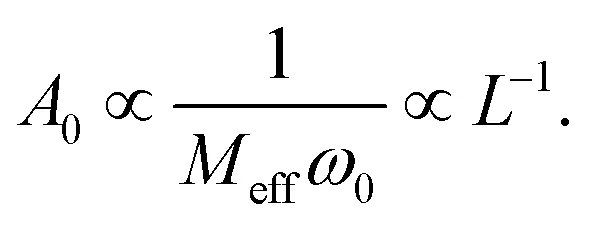


Thus, although increasing the aperture size lowers the resonance frequency, the simultaneous increase in modal mass dominates the single-impact response, and smaller apertures are favourable. This trend is directly visible in Fig. 3.

Reducing the membrane tension provides another possible route because

23 *A*_0_ ∝ *T*^−1/2^.

The square-root dependence again provides only limited leverage: an enhancement of *A*_0_ by a factor *g* would require the membrane tension to be reduced by a factor *g*^2^. Large improvements therefore rapidly require tensions far below the regime assumed for a stable tension-dominated suspended membrane.

### 3.3 Influence of mechanical damping

The quality factor has little influence on the initial single-impact amplitude. From eqn (13),24
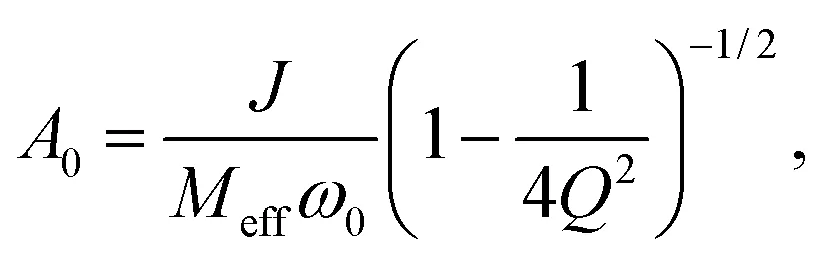


which approaches *J*/(*M*_eff_*ω*_0_) rapidly for *Q* ≫ 1. For the value *Q* = 500 used here, the correction to the undamped amplitude is below 10^−6^. Variations in *Q* therefore primarily change the ring-down time, *τ*_A_ = *Q*/(π*f*_0_), rather than the peak displacement. Even substantial uncertainty in the experimentally achievable quality factor does not alter the predicted single-impact amplitude appreciably.

### 3.4 Improving the optical readout

Another possible strategy would be to improve the optical readout sensitivity. However, the fluorescence detection schemes considered here already operate close to the limits imposed by photon shot noise and realistic fluorescence count rates of nanodiamond emitters.^22^ Even optimistic improvements in optical detection efficiency would therefore only yield modest gains compared to the four orders of magnitude required for single-event detection.

### 3.5 Interferometric readout

An alternative strategy is to detect the membrane displacement interferometrically rather than through fluorescence-intensity modulation. Optical interferometric detection has been demonstrated for graphene mechanical resonators,^26,32^ while cavity-based and integrated optomechanical approaches provide alternative routes to high-sensitivity displacement transduction.^24,25,33^ Representative approaches and reported displacement sensitivities are summarised in Table 1.

**Table 1:** Comparison of displacement-readout approaches relevant to graphene-based nano-mechanical sensing. Reported sensitivities refer to the specific experimental or theoretical conditions of the respective studies and should not be interpreted as directly comparable detection thresholds for the transient single-impact signal considered here

Readout	System	Displacement sensitivity
Optical interferometry^26^	Graphene-SiN_*x*_ hybrid resonator	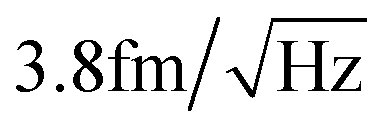
Microwave cavity optomechanics^24^	Multilayer graphene resonator	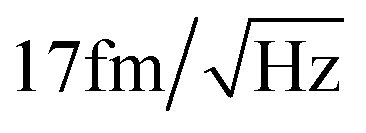
Cavity optomechanics^25^	Multilayer graphene resonator	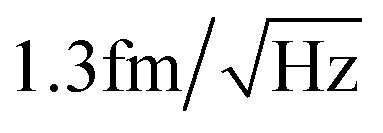
Integrated photonic transduction^33^	Graphene NEMS/silicon photonics	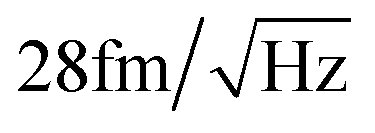 a
Fluorescence modulation (this work)	Graphene-nanodiamond membrane	*A*_0_/*w* < 10^−6^^b^

a Theoretical device study.

b Dimensionless relative fluorescence modulation rather than displacement sensitivity.

In contrast to the fluorescence scheme, the relevant quantity for such a readout is the absolute displacement *A*_0_. The present model predicts single-impact amplitudes ranging from approximately 10^−15^ to 2 × 10^−13^ m over the parameter space shown in Fig. 3. The upper end of this range is therefore comparable in absolute magnitude to the femtometre-scale displacements accessible to state-of-the-art graphene optomechanical measurements. This comparison suggests that interferometric readout is substantially more promising than direct fluorescence modulation.

However, displacement sensitivities quoted in 
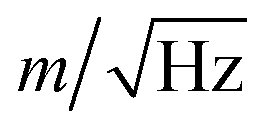
 cannot be compared directly with the peak amplitude of a transient atomic-impact signal. Detectability also depends on the measurement bandwidth, mechanical ring-down time, optical collection efficiency, and the noise spectrum of the complete readout system. The present resonators additionally operate at frequencies of order 10^8^–10^9^ Hz, whereas the optimum sensitivities reported for existing graphene optomechanical devices were obtained for different resonator geometries and measurement conditions. A dedicated noise and bandwidth analysis would therefore be required to establish whether individual atomic impacts can be resolved interferometrically.

Finally, implementing such a readout in a membrane array introduces an additional experimental challenge. Conventional interferometric schemes require optical access to the mechanical element being measured, and individually resolving a large number of apertures would considerably increase the complexity of the optical architecture. Integrated photonic transduction schemes may provide a possible route towards scalable readout,^33^ but their implementation for the membrane geometry considered here remains an open experimental question.

### 3.6 Implications for hybrid nano-mechanical sensors

Taken together, the present analysis establishes quantitative bounds on the use of graphene-nanodiamond membranes for atomic-impact sensing. The mechanical coupling itself is sufficient to generate a well-defined membrane response, but the resulting displacement is extremely small, with single-impact amplitudes in the femtometre to sub-picometre range. For direct fluorescence-intensity readout, the additional normalisation by the optical detection length reduces the relative modulation to below 10^−6^ throughout the investigated parameter space. Combined with photon-counting statistics, this makes single-impact detection through fluorescence modulation impractical under the conditions considered here.

Importantly, this limitation applies to the readout mechanism rather than to the underlying mechanical response. State-of-the-art graphene optomechanical measurements have demonstrated displacement sensitivities in the femtometre-per-root-Hertz range,^24–26^ bringing the upper range of the predicted impact-induced displacements closer to experimentally accessible scales. Whether individual impacts can be resolved by such a scheme, however, depends critically on bandwidth, mechanicalring-down, and the noise characteristics of the complete detection system and requires a dedicated analysis.

The resulting design picture is therefore clear: improving the atomic momentum transfer, membrane geometry, or quality factor provides only limited leverage, whereas the choice of displacement readout is the decisive parameter. The present upper-bound analysis thus identifies direct fluorescence modulation as the principal bottleneck and redirects future development towards substantially more sensitive displacement-transduction schemes. More generally, it provides quantitative design criteria for hybrid nano-mechanical sensors based on two-dimensional membranes and optically active nanostructures.

## 4 Conclusions

In this work, we have analysed a hybrid nano-mechanical sensor concept based on graphene membranes functionalised with fluorescent nanodiamonds. Using a minimal analytical mechanical model, we quantified the resonance properties of suspended graphene membranes and evaluated their response to the momentum transfer associated with single atomic collisions.

Design maps of the mechanical resonance frequency and the resulting oscillation amplitudes were obtained as functions of aperture size, nanodiamond loading, and atomic collision energy. The analysis was deliberately performed under favourable mechanical and collision conditions, such that the calculated response represents an upper-bound estimate within the assumptions of the model.

The predicted single-impact displacement lies in the femtometre to sub-picometre range. When converted into an optical signal through fluorescence-intensity modulation, the corresponding relative modulation *A*_0_/*w* remains below 10^−6^ throughout the investigated parameter space and reaches only a few 10^−7^ under the most favourable conditions. Photon-counting statistics therefore place direct fluorescence detection of individual atomic impacts far beyond the accessible regime for the assumed fluorescence rates.

Real devices introduce additional limitations not included in this upper-bound model. In suspended graphene resonators, fabrication and surface loading can modify not only the effective mass but also the built-in strain and membrane tension, with experimentally observed resonance properties depending sensitively on adsorbates and fabrication history.^20,34^ Nanodiamond attachment may therefore introduce local strain, perturb the mode shape, and provide additional dissipation beyond the homogeneous mass-loading approximation used here. Fabrication of membrane arrays additionally requires reproducible graphene transfer and suspension across the apertures while maintaining membrane integrity and sufficiently uniform mechanical properties across the array.^35^

The graphene-nanodiamond interface introduces an additional optical constraint. Hybrid graphene-nanodiamond devices using NV-centre fluorescence have been realised experimentally, demonstrating that the two components can be integrated into a common nano-electromechanical architecture.^23^ At small graphene-NV separations, however, non-radiative energy transfer to graphene can substantially reduce the NV fluorescence. An energy-transfer efficiency of approximately 40% has been measured for a single NV centre in a nanodiamond coupled to monolayer graphene.^36^ The achievable fluorescence rate can therefore depend on nanodiamond size, NV position, surface chemistry, and the detailed graphene-nanodiamond interface. These effects are not included in the present idealised optical model and would generally further reduce the experimentally accessible fluorescence signal.

The resulting limitation is therefore associated primarily with the fluorescence readout rather than with the absence of a mechanical response. Interferometric and cavity-based graphene optomechanical measurements have demonstrated displacement sensitivities in the femtometre-per-root-Hertz range,^24–26^ bringing the upper range of the predicted impact-induced displacement closer to experimentally accessible scales. Whether individual impacts can be resolved by such schemes remains dependent on measurement bandwidth, mechanical ring-down, and the noise characteristics of the complete detection system.

Overall, the present work establishes quantitative feasibility bounds for atomic-impact sensing with graphene-nanodiamond membranes. Rather than identifying further optimisation of the membrane parameters as a route to single-impact fluorescence detection, the analysis identifies the displacement readout as the central design bottleneck and provides quantitative guidance for future hybrid nano-mechanical sensors based on two-dimensional materials and optically active nanostructures.

## Author contributions

S. E. and J. Z. conceived the sensor concept and proposed the graphene-nanodiamond platform. J. F. developed the theoretical model, performed the calculations, and generated the figures. All authors discussed the results and jointly wrote the manuscript.

## Conflicts of interest

There are no conflicts to declare.

## Data Availability

No experimental data were generated in this study. All numerical results and figures were generated from the analytical model and parameters reported in the article and can be reproduced directly from the equations and parameters provided. No external datasets were used in the analysis
